# *In situ* Microfluidic Cryofixation for Cryo Focused Ion Beam Milling and Cryo Electron Tomography

**DOI:** 10.1038/s41598-019-55413-2

**Published:** 2019-12-13

**Authors:** Marie Fuest, Miroslava Schaffer, Giovanni Marco Nocera, Rodrigo I. Galilea-Kleinsteuber, Jan-Erik Messling, Michael Heymann, Jürgen M. Plitzko, Thomas P. Burg

**Affiliations:** 1 Max Planck Institute for Biophysical Chemistry, Am Fassberg 11, 37077 Göttingen, Germany; 2 Technische Universität Darmstadt, Merckstrasse 25, 64283 Darmstadt, Germany; 3 Max Planck Institute of Biochemistry, Am Klopferspitz 18, 82152 Martinsried, Germany; 4 Institute of Biomaterials and Biomolecular Systems, University of Stuttgart, Pfaffenwaldring 57, 70569 Stuttgart, Germany

**Keywords:** Engineering, Cryoelectron microscopy

## Abstract

We present a microfluidic platform for studying structure-function relationships at the cellular level by connecting video rate live cell imaging with *in situ* microfluidic cryofixation and cryo-electron tomography of near natively preserved, unstained specimens. Correlative light and electron microscopy (CLEM) has been limited by the time required to transfer live cells from the light microscope to dedicated cryofixation instruments, such as a plunge freezer or high-pressure freezer. We recently demonstrated a microfluidic based approach that enables sample cryofixation directly in the light microscope with millisecond time resolution, a speed improvement of up to three orders of magnitude. Here we show that this cryofixation method can be combined with cryo-electron tomography (cryo-ET) by using Focused Ion Beam milling at cryogenic temperatures (cryo-FIB) to prepare frozen hydrated electron transparent sections. To make cryo-FIB sectioning of rapidly frozen microfluidic channels achievable, we developed a sacrificial layer technique to fabricate microfluidic devices with a PDMS bottom wall <5 µm thick. We demonstrate the complete workflow by rapidly cryo-freezing *Caenorhabditis elegans* roundworms L1 larvae during live imaging in the light microscope, followed by cryo-FIB milling and lift out to produce thin, electron transparent sections for cryo-ET imaging. Cryo-ET analysis of initial results show that the structural preservation of the cryofixed *C*. *elegans* was suitable for high resolution cryo-ET work. The combination of cryofixation during live imaging enabled by microfluidic cryofixation with the molecular resolution capabilities of cryo-ET offers an exciting avenue to further advance space-time correlative light and electron microscopy (st-CLEM) for investigation of biological processes at high resolution in four dimensions.

## Introduction

Cryo-electron microscopy (cryo-EM) has recently emerged as a method to analyze the structure of biological macromolecules with near atomic resolution^1,2^. Cryo-electron tomography (cryo-ET) further allows 3D reconstruction of individual macromolecules in their cellular context from a tilt series of cryo-EM images, providing a unique avenue to study the molecular mechanisms that drive biological processes. Cryofixation is the foundation of cryo-ET. Classical room temperature electron microscopy (EM) studies of biological samples rely on dehydration, chemical fixation, and plastic embedding^3^ to render samples compatible with the high vacuum of the electron microscope. The sample preparation procedures, and the dehydration step in particular, often lead to artefacts and distortions^4–7^ that limit structural interpretations.

By contrast, cryo-EM images frozen, hydrated samples in a near native state below the glass transition temperature of water. The quality of structural preservation achieved in cryofixation depends critically on the ability to freeze the samples into an amorphous, or vitrified, state without ice crystallization. This can be accomplished by freezing at high rates (~10^4^ °C/s or higher^8–10^). For samples thicker than several micrometers, high pressure freezing (HPF) must be applied. HPF dates back to the late 1960s^11,12^ and still remains the only method available for cryofixation of whole cells or small organisms as thick as ~150 µm^8^.

An inherent restriction of cryo-EM is that it gives only a snapshot of a biological process at a single time point. To understand how ultrastructural changes observed by electron microscopy relate in time to dynamic cell function, it is thus of great interest to be able to correlate high resolution cryo-electron micrographs with live cell imaging just before cryofixation. Currently, the required sample transfer from the light microscope to the high pressure freezer limits this ability^13–15^. Current automated transfer systems require approximately one second for the transfer from live imaging to high pressure freezing^16^. Innovative studies have worked to bypass this limitation by initiating a desired reaction with a light or electrical stimulus and carefully timing or programming cryofixation to occur a few to tens of milliseconds after the external trigger^10,17–21^. These methods, however, do not yet allow uninterrupted live imaging of the dynamic process up to cryofixation.

We have recently shown a microfluidic based technology that is able to cryofix samples directly in the light microscope^22^. This technology enables millisecond time correlation between live imaging and electron microscopy, where the time correlation is limited only by the sample freezing time. This technique is of interest for studying rare, highly dynamic, or directional processes by real time selection and arrest of a desired biological state. Examples of such processes exist in many areas of cell biology, such as intracellular transport^23^, membrane trafficking, motility, cell division, or immune cell activation.

To date, microfluidic cryofixation has been limited to room temperature electron microscopy studies following freeze substitution and resin embedding of cryofixed samples^22^. Accessing the native *in situ* cellular structures with high resolution cryo-ET was a substantial challenge due to the inability to form electron transparent cryo-sections from samples embedded in the microchannels.

Traditionally, cryo-ultramicrotomy (−140 °C to −160 °C) has been used to create thin (<300 nm) sections from cryofixed intact cells or small organisms using a diamond blade^24,25^. Cryo-ultramicrotomy, however, cannot be easily adapted for samples embedded in microfluidic devices. Variability in material density, stiffness, and cohesion of the different device layers hinder cutting of homogenously thin, continuous cryo-sections without mechanical deformations. A promising alternative approach involves milling samples under cryo-conditions with a focused ion beam (FIB)^26–28^. The combination of cryo-FIB sample preparation with advanced transmission electron microscopy (TEM) methods^29–33^ has recently enabled imaging and 3D reconstruction of macromolecular complexes in their native cellular context with molecular resolution^34,35^.

The final resolution of the cryo-ET results, however, depends on the quality of the prepared cryo-sections, or lamellas. Ideal lamellas are homogeneously thin, free of surface contamination, and free of artefacts such as redeposited material or devitrification^27^. Excessive cryo-FIB milling can lead to degradation of lamella quality, limiting the maximum size of the specimen that can be prepared for cryo-ET^28,36^.

In earlier designs, fabrication constraints limited the dimensions of the PDMS microchannel cross section to ~15 µm × 50 µm × 30 µm bottom, side, and top walls respectively. Given this geometry, removal of ~10^4^ µm^3^ of PDMS (see Fig. S2 and Table S1 for milling geometry) would be required for preparing a lamella. This was not practically feasible due to the extremely long milling time (~40 hours) and the associated sample degradation. Therefore, connecting microfluidic cryofixation with cryo-ET via cryo-FIB lift out required a new microfluidic device technology.

Here we demonstrate an *in situ* microfluidic cryofixation method for connecting live cell imaging with cryo-ET via recently reported cryo-FIB/lift out procedures^28^ for frozen hydrated lamellae. Here, we developed a sacrificial layer technique that enables fabrication of devices with bottom walls at the single micron scale with accurate, sub-micron thickness control. Decreasing the PDMS bottom wall thickness to <5 µm was a critical step for adapting this cryofixation method for cryo-ET. The volume of the polymer wall that had to be milled away was reduced from ~10^4^ µm^3^ to the order of 10^3^ µm^3^, allowing us to prepare electron transparent cryosections of the sample embedded in the microchannel.

To establish the feasibility of this approach, *in situ* cryofixed samples were imaged for the first time using cryo-ET. As a model system, we selected the roundworm *Caenorhabditis elegans* (*C*. *elegans*), which has been a popular model system for neuronal, behavioral, and genetic studies for more than thirty years^37–39^. *C*. *elegans* is well suited for investigations that link the underlying molecular mechanisms to animal behavior, such as mapping neuronal signaling in response to stimuli^40,41^ and correlative light and electron microscopy studies^42,43^. Our initial results show that L1 larvae of *C*. *elegans* can be cryofixed during continuous live imaging in the light microscope and prepared for cryo-ET via cryo-FIB lift out without deformations or evidence of crystalline ice damage in the frozen tissue. The single micron thickness of the PDMS leads to a ~10x increase in cooling rate compared to previously reported designs of the microfluidic cryofixation system^22^. This work paves the way for promising future investigations that combine the millisecond time resolution of microfluidic cryofixation with the *in situ* structural analysis at molecular resolution provided by cryo-ET.

## Results

### Microfluidics enables *in situ* cryofixation

**Figure 1:**
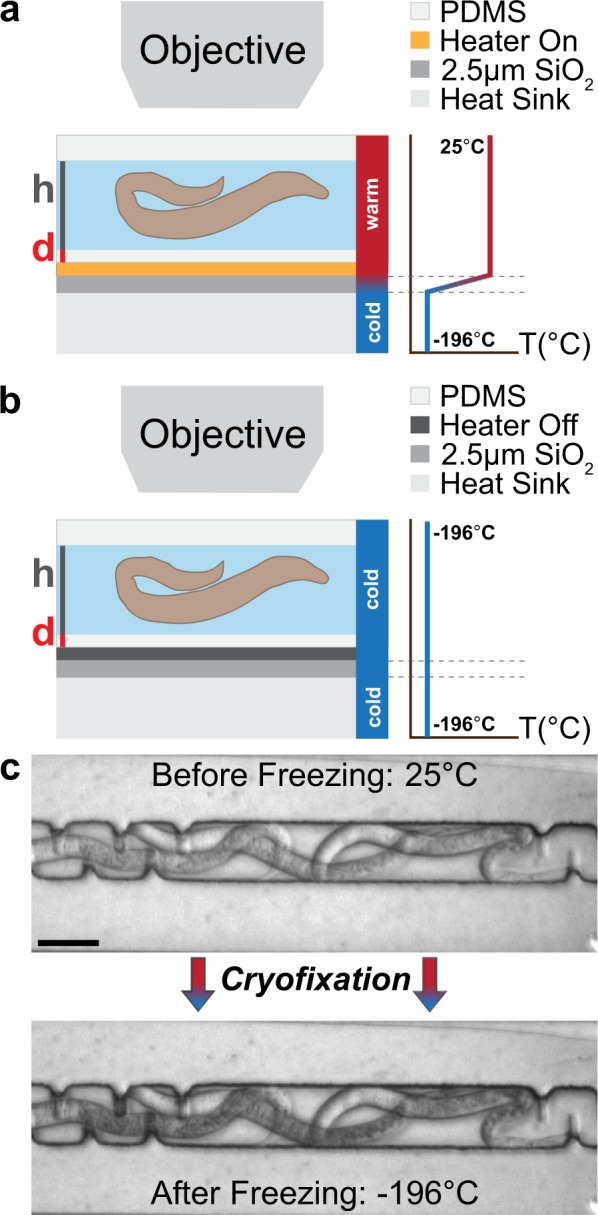
Operating principle of the *in situ* microfluidic cryofixation system. The characteristic length scale of microfluidics makes the system a viable tool for cryofixation. (**a**) A PDMS microchannel is mounted onto a thin film resistive heater. The heater is placed in thermal contact with a LN_2_ cooled heat sink. During live imaging, the power applied to the heater balances the heat loss to the heat sink, maintaining a steep temperature gradient between the microchannel (25 °C) and the heat sink (−196 °C). (**b**) When the heater is turned off, the channel is cryofixed within the microscope field of view. (**c**) *C*. *elegans* shown before and after cryofixation. The *C*. *elegans* were suspended in M9 solution with 10% (m/v) glycerol added as a cryoprotectant. Scale bar: 30 µm.

The basic principle of the *in situ* microfluidic cryofixation method relies on conductive heat transfer from a liquid nitrogen (LN_2_) cooled heat sink (Fig. 1a). The characteristic length scale of microfluidics makes this approach to cryofixation feasible by (i) limiting the total distance from the sample to the cold surface to the order of microns, and (ii) minimizing the thermal mass of the system and thus the thermal energy that must be dissipated during cryofixation^10,44,45^.

A PDMS (polydimethylsiloxane) microchannel, that houses the sample of interest, is mounted onto a thin film resistive heater (Fig. 1). The heater is placed in thermal contact with a LN_2_ cooled heat sink. During live imaging, the power applied to the heater balances the heat loss to the heat sink. The heater maintains a steep temperature gradient between the microchannel (25 °C) and the heat sink (−196 °C) that drops across a 2.5 µm SiO_2_ insulation layer. At the desired moment, the power to the heater is turned off and the contents of the channel cool rapidly via thermal conduction at atmospheric pressure. The experimentally measured cooling rate averaged throughout the channel depth is 2·10^4^ °C/s (see below). A numerical model of the cooling rate as a function of position along the microchannel depth, PDMS thickness, and channel height can be found in Mejia *et al*.^45^.

An example of *C*. *elegans* before and after cryofixation within the microscope field of view is shown in Fig. 1b. The microchannel temperature was first calibrated to 25 °C according to previously reported procedures^22^. *C*. *elegans* suspended in M9 solution with 10% (m/v) glycerol were injected into the microchannel just prior to cryofixation. A 100 fps video showing the arrest of live *C*. *elegans* via cryofixation is given in the Supporting Information.

**Figure 2:**
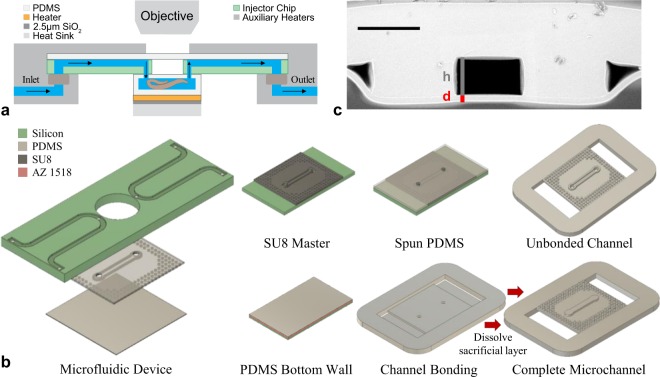
Microfluidic device for *in situ* cryofixation. (**a**) The fluidic network consists of three components: macroscale fluidic channels (inner diameter: 0.8 mm), a silicon injector chip, and the main PDMS microchannel that houses the sample. Auxiliary heaters warm the tubing and injector chip to ensure flow until the moment of cryofixation. The main microchannel is designed with insulating air spaces surrounding the top and sidewalls in order to minimize the relevant thermal mass for cooling. A schematic of the fully assembled cryofixation system can be found in the Supporting Information. (**b**) Schematic of the fabrication procedure used to reliably produce PDMS microchannels with a bottom wall thickness *d* < 5 µm. Briefly, an SU8 master serves as the negative pattern of 20 µm deep microchannels with fluidic through holes. Uncured PDMS is spun onto the master and bonded to a PDMS handling frame, allowing simultaneous fabrication of all 28 microchannels. To form the bottom wall, a sacrificial photoresist layer (AZ1518) was spun onto a silicon wafer, followed by a layer of uncured PDMS. Unsealed channels were bonded to the PDMS bottom via oxygen plasma bonding. The sacrificial layer was then dissolved in acetone to release the channels and complete fabrication. Further details on the fabrication process are given in the Supporting Information. (**c**) A cross section SEM image of a device fabricated according to the procedures outlined in b. The channel height and bottom wall are 20 µm and 2.9 µm respectively. Scale bar: 30 µm.

A unique feature of this microfluidic based cryofixation system is that fluid flow remains possible until the moment of cryofixation, opening the possibility to manipulate the experimental conditions while monitoring the effects on the sample with the light microscope (see Fig. S1 for a full system schematic). The main microchannel forms part of a three-component fluidic network (Fig. 2a). Macroscale fluidic channels connect to a silicon injector chip, used to introduce the sample into the PDMS microchannel.

The sample is visible within the PDMS microchannel through a 1 mm diameter view window etched into the silicon injector chip. The view window provides optical access for imaging and introduces a thermally insulating air space above the sample. The microchannel forms part of a 5 mm × 5 mm PDMS foil with an array of microposts surrounding the main microchannel. The microposts introduce an air space around the microchannel sidewalls and provide mechanical stability that prevents excessive loading of the channel. With three thermally isolated boundaries, the relevant thermal mass for cryo-cooling is thus limited to the PDMS microchannel (PDMS bottom wall, medium filled channel, and PDMS top wall). The PDMS microchannel height is adjusted to the sample size. Here we have chosen a channel height of 20 µm to accommodate typical bending behavior of L1 stage *C*. *elegans* (body diameter ~12 µm). Considering the channel height constraint and the significantly lower FIB milling rate for PDMS compared to the sample/medium, the thickness of the PDMS bottom wall (*d*) is the critical parameter that determines both the FIB milling time and the system cooling rate.

### Defining a < 5 µm critical dimension

To achieve the required *d* < 5 µm, a sacrificial layer technique combined with soft lithography was developed to decouple bottom wall fabrication from the PDMS microchannel fabrication (Fig. 2b). A two-step photolithography procedure was used to create a negative of the microchannels in SU8 with aligned microposts in the reservoirs as the negative pattern of fluidic through holes. Liquid PDMS was spun over the master and cured. Surface tension of the liquid PDMS around the microposts leads to local thickening of the PDMS layer near the through holes. The spin coating recipe minimizes the height difference between the spun PDMS layer and the microposts to limit local nonplanarity and subsequent incomplete bonding in the final devices. Here, the SU8 molding process forms the microchannel sidewalls and top wall, which has through holes for fluidic access.

Here we develop a new approach for simultaneous fabrication of 28 PDMS microchannels. Briefly, a PDMS handling frame of 100 mm diameter and 2 mm thickness was formed and laser cut with access points for each microchannel. The handling frame was bonded to the PDMS coated master using partial cure bonding^46^. Peeling the frame from the master produces 28 suspended membranes, each containing a three walled PDMS microchannel.

To create the channel bottom, a sacrificial ~2 µm thick positive tone photoresist layer (AZ 1518) was first spun onto a silicon wafer. Uncured 10:1 PDMS was spun directly on top of the sacrificial layer, where the selected spin coating parameters set the final thickness of the bottom wall. The 28 open microchannels were then bonded to the spin coated PDMS layer using oxygen plasma bonding to form sealed channels. After bonding, the sacrificial AZ 1518 layer was dissolved away using acetone. The sacrificial layer technique allows consistent fabrication of microfluidic devices with a bottom wall *d* < 5 µm thick. Figure 2c shows a cross section image of a representative device with *h* = 20 ± 1 µm and *d* = 2.9 ± 0.2 µm. Full details on the microchannel fabrication procedure are given in the Supporting Information.

### Cooling rate characterization

A reduction in the microchannel bottom wall thickness from ~15 to 3 µm significantly increases the cooling rate in the microchannel, which ultimately governs the quality of the sample preservation. The cooling rate in the microchannel is primarily determined by the thickness of the PDMS microchannel bottom wall (*d*) and the height of the channel (*h*) With a thermal conductivity of 0.15 W/mK for PDMS, about 4x lower than that of water (0.6 W/mK at 20 °C), reducing the thickness of the PDMS bottom wall has a more significant effect on the cooling rate than an equivalent reduction in the channel height (i.e. depth of the aqueous medium). Here we experimentally measure the time required for the contents of the channel to cool from the working experimental temperature (25 °C) to ~−21 °C, by monitoring the fluorescence intensity of a Rhodamine B/DI water solution during cryofixation.

Rhodamine B is a commonly used probe for non-contact temperature measurements as its quantum yield, and subsequently fluorescence intensity, increases as the medium cools^47–49^. One recent report demonstrated Rhodamine B/DI water solution as a probe for the solid-liquid phase transition^50^. Fluorescence intensity of the Rhodamine B/DI water solution increased as the solution approached the freezing point, reaching a maximum just before the solid-liquid phase transition. A similar effect was observed during slow, controlled cooling of our microfluidic cryofixation system (see SI). Briefly, the heater power was slowly decreased in steps, with each step corresponding to a 1 °C change in the equilibrium channel temperature. Fluorescence intensity increased correspondingly until reaching a maximum just before the solid-liquid phase transition.

**Figure 3:**
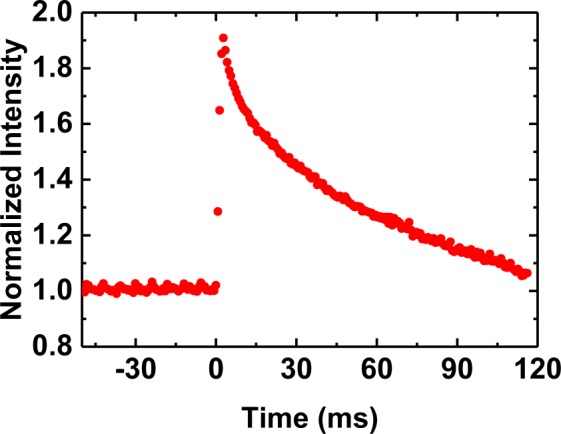
Rhodamine intensity was normalized to the intensity at 25 °C, the initial temperature of the solution. During *in situ* cryofixation a fluorescence maximum is reached within 2.4 ms. Based on the known dependence of fluorescence intensity between 25 °C to ~−21 °C, the initial cooling rate averaged over the channel depth was 2·10^4^ °C/s. Photophysical effects dominate the measured intensity after the maximum is reached.

Figure 3 shows the fluorescence intensity of the Rhodamine B/DI water solution acquired during *in situ* microfluidic cryofixation by rapid cooling. The channel temperature was initially held constant at 25 °C. From videos with a frame rate of 1250 fps or higher, we measured a time of 2.4 ± 0.4 ms from the moment the heater was turned off until the fluorescence maximum was reached. Based on the known temperature dependence of Rhodamine intensity between 25 °C and −21 °C, the cooling rate averaged throughout the channel depth was, to first order, 2·10^4^ °C/s.

The Rhodamine measurement sets a lower bound for the initial cooling rate. Note that the observed fluorescence decay following the intensity maximum in Fig. 3 can be explained solely by photophysics rather than by a temperature change. To test this hypothesis, we switched off the fluorescence illumination intermittently and found that the initial peak brightness was fully restored after leaving the sample in the dark for a few seconds (Fig. S6). We therefore explain the gradual decay of fluorescence in the frozen sample during illumination by the population of nonfluorescent electronic states (dark states), which can be relatively long lived under cryo-conditions^51,52^.

### Bridging microfluidic cryofixation and cryo-transmission electron microscopy

**Figure 4:**
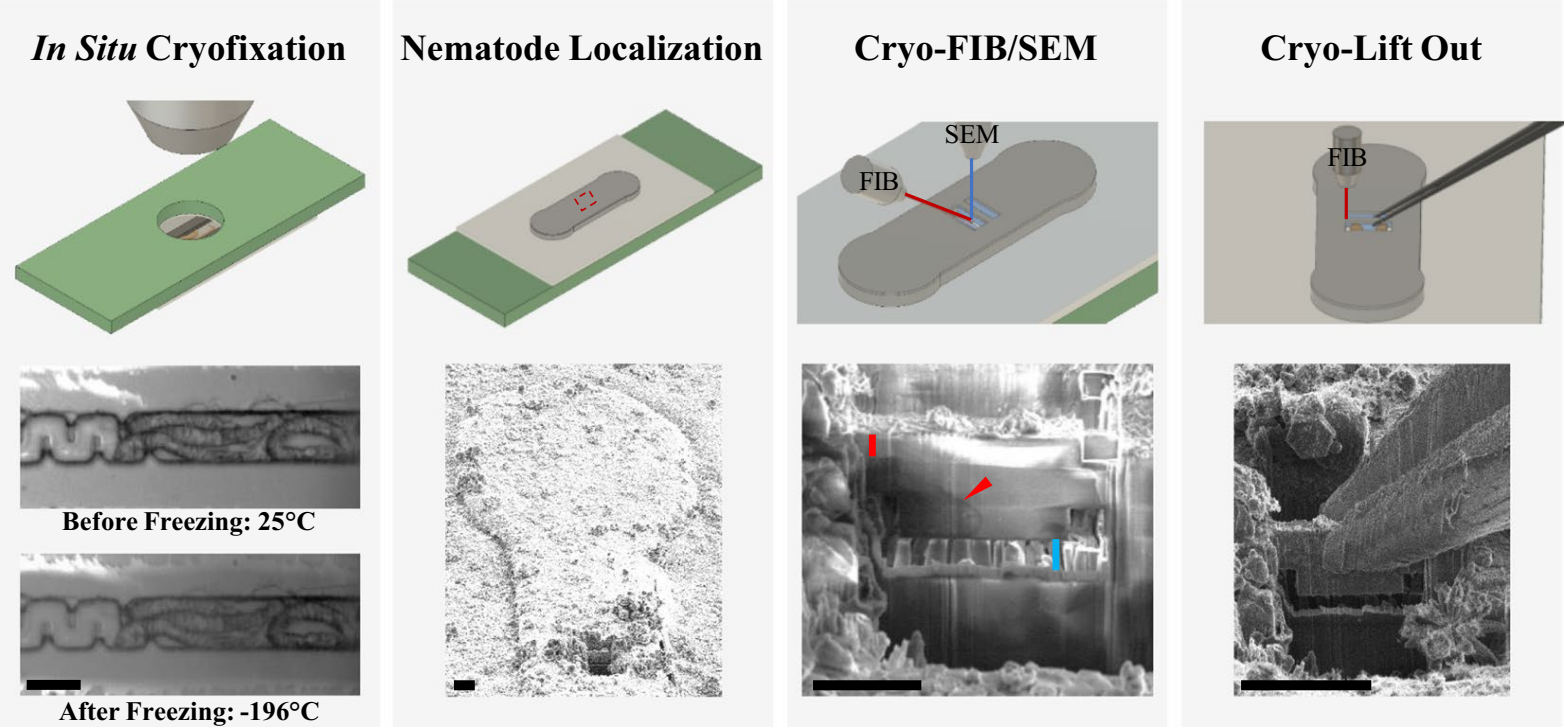
Bridging *in situ* microfluidic cryofixation and cryo-ET. ***In Situ*** **Cryofixation**: A live *C*. *elegans* was cryofixed within the light microscope field of view using the microfluidic based system. The *C*. *elegans* was suspended in M9 with 10% (m/v) glycerol added as a cryoprotectant. Scale bar: 30 µm. **Nematode localization**: The outline of the protruding microchannel was clearly visible in the scanning electron microscope. Bright field images of the nematode location following cryofixation were referenced to known channel geometry to locate the nematode. Deposition of frost contamination from ambient humidity during sample transfers is evident on the external surface of the microfluidic device. Scale bar: 20 µm. **Cryo FIB/SEM:** The region of interest was milled with a focused ion beam to form a 4.5 µm thick lamella. The PDMS channel bottom is marked by a red line in the SEM image (length 3 µm). The frozen hydrated channel interior extends from the end of the red line towards the undercut (blue line). Scale bar: 10 µm. **Cryo Lift Out:** A cryo-gripper micromanipulator was used to remove the lamella from the microchannel. Scale bar: 20 µm.

As a proof of feasibility, *in situ* microfluidic cryofixation was connected for the first time to cryo-ET using cryo-FIB milling and a cryo-lift out^53,54,55^ procedure (Fig. 4). The first panel in Fig. 4 shows light microscope images of multiple *C*. *elegans* in the microchannel before and after freezing. *C*. *elegans* was suspended in M9 with 10% (m/v) glycerol as a cryoprotectant. Traces of most likely the *E*. *coli* lawn from the nematode growth plates can be seen in the medium. Following cryofixation, the microfluidic device was dismounted from the *in situ* cryofixation system under LN_2_ according to previously reported procedures^22^ and transferred to the cryo FIB/SEM.

The outline of the protruding microchannel walls can be seen in the scanning electron microscope image (Fig. 4, panel 2). In this proof of principle investigation, the nematode was localized manually by comparing bright field images of its location following cryofixation to the known overall microchannel geometry (see SI). In future work, we intend to adapt existing methods^56^ in order to automatically localize regions of interest in the microchannel sample for cryo-FIB milling.

A 4.5 µm thick lamella was then formed by milling orthogonal to the 3 µm PDMS channel bottom on both sides of the region of interest. The sample was then tilted and rotated to have a 21° milling angle with respect to the sample surface to undercut the lamella in preparation, connect the previously milled trenches, and thereby remove the lamella from the microchannel (Fig. 4). The location of the undercut and the 3 µm thick PDMS layer are marked by the blue and red bars respectively. The total PDMS milling volume including the undercut was on the order of 10^3^ µm^3^ (see SI). The red arrow indicates the location of the nematode within the milled lamella (Fig. 4). From the SEM images, up to ~6 µm of medium separated the nematode from the bottom wall of the channel during cryofixation.

A dedicated cryo-gripper micromanipulator then removed (“lifted out”) the ~15 µm wide × 15 µm deep × 4.5 µm thick lamella from the microchannel (Fig. 4). After gripping the lamella with the micromanipulator, the edges of the lamella were milled with the FIB to disconnect it from the microchannel. The lamella was then transferred to a prepared slot in a TEM half grid and fixated by non-localized organometallic Pt deposition, where it was thinned with stepwise decreasing beam current to the final thickness (~100 nm) for TEM imaging.

## Discussion

### Cryo-EM of *in situ* cryofixed *C*. *elegans*

**Figure 5:**
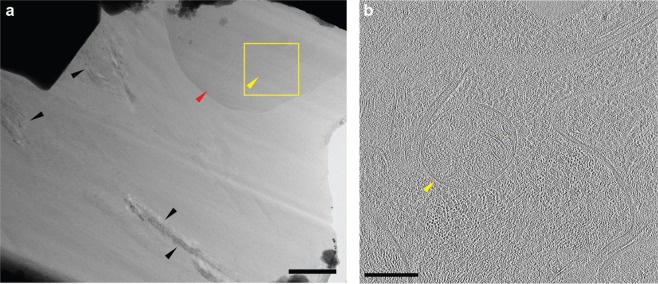
Cryo-transmission electron microscopy of an *in situ* cryofixed *C*. *elegans*. The nematode was suspended in M9 with 10% (m/v) glycerol added as a cryoprotectant. (**a**) Cryo-TEM overview. Some ice crystallization was observed in the M9/glycerol medium (black arrows) but not within the nematode (red arrow). The yellow rectangle and yellow arrow indicate the location of the magnified tomographic reconstruction shown on the right. Scale bar: 1 µm. (**b**) Slice from the tomographic volume reconstructed from the cryo-ET tilt series of an *in situ* cryofixed *C*. *elegans*. The tomogram slice has the same orientation as the overview image to the left and corresponds to the yellow rectangle. No artefacts from ice crystallization are observed within the tissue. Scale bar: 200 nm.

After an initial check of sample quality with low magnification cryo-TEM imaging, a tilt series at higher magnification was acquired and a tomographic reconstruction computed therefrom. Figure 5a shows an overview cryo-TEM image of the lamella following lift out and thinning to ~100 nm. No artefacts from ice crystallization were observed within the *in situ* cryofixed *C*. *elegans* tissue in either the overview image (Fig. 5a) or, more importantly, the slice through the reconstructed tomogram (Fig. 5b) of the nematode. The overview image does, however, show some ice crystallization within the M9/glycerol medium surrounding the nematode, as indicated by the black arrows. Ice crystallization can occur from a variety of sources including (i) insufficient cooling rate during cryofixation (ii) insufficient heat dissipation leading to devitrification during FIB milling, and (iii) sample warming during (multiple) required transfers between instruments. Detailed investigations are required to determine the cause of the observed ice crystallization and are the subject of future work.

### Cooling rate and limitations

One fundamental limitation of the microfluidic cryofixation is the size of the samples that can be cryofixed for cryo-ET. The cooling rate of the system determines this limit. When the sample cooling rate is insufficient for vitrification throughout the sample thickness, intracellular water forms crystalline ice, leading to local segregation, concentration of biological material, and distortion of the native architecture^8,9,57^. For the present system with 20 µm tall microchannels and a 3 µm PDMS channel bottom, we measured an initial cooling rate of 2·10^4^ °C/s, in line with reported cooling rates for other cryofixation methods at atmospheric pressure^9,10^. PDMS bottom walls down to 0.8 µm thick have been fabricated with the protocols outlined here. However, further reducing the bottom wall from 3 µm to 0.8 µm gives only a modest increase in cooling rate of ~1.5 × (see SI).

The sacrificial layer technique decouples the bottom wall fabrication from the fabrication of the unsealed microchannel and, therefore, could be applied to materials of higher thermal conductivity to further increase the cooling rate. Silicon nitride membranes and many other ceramics, have nearly three orders of magnitude better thermal conductivity than water. If used as a channel bottom, the influence of such membranes on the cooling dynamics could be reasonably neglected and the cooling rate would be determined by the sample itself and any surrounding medium.

*In situ* microfluidic cryofixation faces the same sample size limitations known for cryofixation at atmospheric pressure. The thermal conductivity of water limits the achievable cooling rate within the sample, which declines with increased depth inside the sample, thereby limiting the achievable vitrification depth. Experimental results for slam freezing, which also relies on one sided heat conduction at atmospheric pressure, report preservation for biological samples of up to ~10–15 µm without the addition of cryoprotectants^9,10,58^. We expect that limitations for *in situ* microfluidic cryofixation with optimized geometry and materials will likely fall within a similar range^22^. Achievable results, however, vary widely across samples as the critical cooling rate depends on the natural water/solute content^8,58,59^, where higher concentrations of proteins/solutes in the sample act as natural cryoprotectants. Thickness limitations for a given sample are thus difficult to predict.

Cryoprotectants can be added to relax the cooling rate requirement for vitrification, though care must be taken to avoid interfering with the biological question of interest. In some circumstances, cryoprotectants can reduce the critical cooling rate of the aqueous solution by ~100x or more^60,61^, where the critical cooling rate is an exponential function of cryoprotectant concentration^60^. A reduced rate requirement of ~100x corresponds to a ~10x increase in vitrification depth^8^, as expected for diffusion processes where diffusion time is proportional to the distance squared.

Due to the variation in water/solute content, the critical cooling rate and thus the preservation quality for the sample and surrounding medium can vary significantly. Ice crystals that form in the dilute medium may promote crystallization at some locations within the sample itself. However, the extent to which the quality of the sample preservation may be affected by the freezing quality in the surrounding medium is likely sample and cryoprotectant dependent.

We expect that by increasing the cooling rate above 10^4^ °C/s, and thus reducing the required cryoprotectant concentration, we have expanded the applicability of *in situ* microfluidic cryofixation to a wider range of samples. Appropriate cryoprotectant type and the maximum concentration that may be used depends on toxicity and tolerance to osmotic stress, which varies across samples. Here, glycerol was chosen based on toxicity and motility assays performed for 8 different commonly used cryoprotectants at two different concentrations (16 total samples, See SI). Ultimately, individual sample size, natural solute concentration, and tolerance to cryoprotectants will determine if the ultrastructural preservation achieved using the *in situ* microfluidic cryofixation technique is sufficient for high resolution cryo-electron tomography studies of a specific sample.

## Conclusions

In this work, we demonstrate cryofixation during live imaging followed by cryo-transmission electron tomography. The roundworm *C*. *elegans* was used as a model system. To enable cryo-FIB preparation of electron transparent lamellae from *C*. *elegans* cryofixed within microfluidic channels, we devised a fabrication process that yields microchannels with a 0.8 ± 0.1 µm to 2.9 ± 0.2 µm thin bottom wall. The total cryo-FIB milling volume of the PDMS is thereby reduced to the order of 10^3^ µm^3^, which is critical to avoid excessive milling times and consequent deterioration of the sample during trench preparation.

With a 3 µm critical dimension, the measured initial cooling rate during microfluidic cryofixation in the light microscope was >10^4^ °C/s, an ~10x improvement compared to previous designs of the *in situ* microfluidic cryofixation system. The minimum cryoprotectant concentration for sample ultrastructure preservation decreases with higher cooling rates. We therefore expect the higher cooling rate to increase the applicability of this cryofixation technique to a wider range of samples. The fabrication process is not limited to PDMS channel bottoms and can be extended to a variety of materials with higher thermal conductivity, such as silicon nitride, to further increase the cooling rate.

As a proof of feasibility, *in situ* cryofixed microfluidic samples were imaged for the first time using cryo-transmission electron microscopy. Cryo-ET results show no artefacts from ice crystallization within the *Caenorhabditis elegans*. The quality of the sample preservation was suitable for high resolution cryo-ET work. Initial results suggest a promising future that connects the millisecond time correlation capabilities of *in situ* microfluidic cryofixation with the molecular spatial resolution of cryo-ET in order to study biological processes in four dimensions.

## Materials and Methods

*C*. *elegans* strain HBR4: goeIs3[pmyo-3::GCamP3.35::unc-54–3’utr, unc-119(+)]V, expressing the green fluorescent protein calcium indicator GCaMP3.35^62^, was used as a test sample. *C*. *elegans* were grown on nematode growth medium (NGM) agarose plates seeded with *E*. *coli* OP50 and maintained at 20 °C^62^. The *C*. *elegans* were suspended in M9 buffer with 10% (m/v) glycerol (Invitrogen) as a cryoprotectant and then introduced into the microchannel. The NiCr resistive heaters were fabricated similar to previously reported procedures^45^. A full description of the microfluidic device fabrication procedures is given in the Supporting Information.

Light microscopy images were taken using a 10x air objective (0.3 NA) with a Nikon E-600 microscope and a Thorlabs LED Source. The cooling rate measurement was performed using DI water with 1% (m/m) Rhodamine B isothiocyanate–Dextran (RhB-ITC-Dextran 70 kDa, Sigma-Aldrich) to prevent penetration of the Rhodamine solution into the PDMS walls. The microchannel temperature was initially set to room temperature according to calibration procedures described previously in detail^22^. Fluorescence videos were acquired with an Andor Neo sCMOS camera at a frame rate of 1250–1593 fps using a mercury lamp as a light source (IntensiLight, Nikon), a 546/10 nm excitation filter, and a 575 nm long pass emission filter.

### Methods for cryo-FIB

All cryo-FIB work was carried out on a Dual-beam FIB Quanta 3D FEG (Thermo Fischer Scientific, Eindhoven, NL) equipped with an in house built cryo-stage and a Quorum PP3000T cryo-system (Quorum Technologies, Laughton, United Kingdom), which includes cooling control, a cooled anti-contaminator, a transfer unit and an attached cryo-preparation chamber. The cryo lift out technique utilized the cryo-gripper tip mounted on the MM3A-EM micromanipulator (Kleindiek Nanotechnik GmbH, Reutlingen, Germany) which is cooled by thermal connection to the Quorum anti-contaminator.

The frozen microfluidic chip was first mounted into the FIB shuttle and transferred into the Quorum prep chamber, where it was sputter coated with Pt for 45 sec (at 10 mA) to improve conductivity. It was then transferred onto the cryo-stage of the FIB chamber. Using the gas injection system (GIS) of the FIB, a protective organometallic Pt layer was deposited over the full specimen surface (GIS temperature 26 °C, gas flow opened for 25 sec at a needle distance of 3.5 mm). A thick lamella (15 × 15 × 4.5 µm) was produced with the ion beam (30 kV beam voltage; sequentially decreasing ion beam currents of 3 nA, 1 nA, and 0.5 nA; regular rectangular scanning pattern) using standard procedures^63^. After cryo-transfer with the gripper and mounting on a TEM half grid within the same FIB chamber, the lamella was thinned to final thickness of ~100 nm following established cryo-preparation protocols^27,64^ (30 kV beam voltage; sequentially decreasing ion beam currents of 0.3 nA, 0.1 nA, and 30 pA; regular rectangular scanning pattern). The finished TEM sample holding the lamella was then transferred under cryo-conditions from the FIB chamber into liquid Nitrogen storage. A more detailed description of the cryo-lift out procedure is described by Schaffer *et al*.^28^

### Methods for cryo-ET

Data acquisition was done using the SerialEM software^28,65^ at 300 kV on a Titan Krios (Thermo Fischer Scientific, Eindhoven, NL) equipped with a Quantum post column energy filter (Gatan, Pleasanton, CA, USA) and a K2 Summit direct detector camera (Gatan, Pleasanton, CA, USA). The tilt series was acquired with a pixel size of 0.342 nm, a target defocus of −5 µm, and using dose fractionation mode at 12 frames per second. Individual exposure times between 0.6 and 2.5 sec were automatically adjusted by the software to compensate for thickness variation during tilting. The total dose over the full series was kept below 100 e^−^/Å^2^. Data processing and tomogram reconstruction used MotionCor2^66^ and IMOD^67^ software.

## Supplementary information

Supporting Information

C. elegans Cryofixation in the microscope field of view
