# Supplementary material for: In situ Microfluidic Cryofixation for Cryo Focused Ion Beam Milling and Cryo Electron Tomography
Source: Sci Rep. 2019 Dec 13;9:19133. doi: 10.1038/s41598-019-55413-2 (PMC6911106; doi:10.1038/s41598-019-55413-2)
Supplement: Supplementary file 1 — Supporting Information [file 41598_2019_55413_MOESM1_ESM.docx]

*In situ* Microfluidic Cryofixation for Cryo-Focused Ion Beam Milling and Cryo‑Electron Tomography

**Supporting Information**

Marie Fuest^1^, Miroslava Schaffer^3^, Giovanni Marco Nocera^1^, Rodrigo I. Galilea‑Kleinsteuber^1^, Jan‑Erik Messling^1^, Michael Heymann^3,4^, Jürgen M. Plitzko^3^, and Thomas P. Burg*^1, 2^

*^1^Max Planck Institute for Biophysical Chemistry, Am Fassberg 11, 37077 Göttingen, Germany*

*^2^Technische Universität Darmstadt, Merckstrasse 25, 64283 Darmstadt, Germany*

*^3^Max Planck Institute of Biochemistry, Am Klopferspitz 18, 82152 Martinsried, Germany*

*^4^Institute of Biomaterials and Biomolecular Systems, University of Stuttgart, Pfaffenwaldring 57, 70569 Stuttgart, Germany*

*Corresponding Author

ABSTRACT

The Supporting Information contains a caption for the Supplementary video, additional information on the fully assembled *in situ* cryofixation system, and an estimate of the cryo-FIB milling volume required to produce a lamella from the microchannel. Details of the microfluidic device fabrication are described. Further details of the cooling rate measurement are given, including cooling rate measurements for microchannels with a bottom wall of 0.8 µm thickness. Additional slices from the tomogram taken within the *C. elegans* as well as the images from the full tilt-series used for the volume reconstruction are given. Further information is presented on cryoprotectant toxicity for *C. elegans* and the effect of cryoprotectants on motility.

CRYOFIXATION VIDEO

The video shows cryofixation of a live *C. elegans* within the microscope field of view captured at 100 fps. The *C. elegans* was suspended in M9 with 10% (m/v) glycerol as a cryoprotectant. The video was taken with an Andor Neo sCMOS camera and a 10x Nikon air objective (0.3 NA).

IN SITU MICROFLUIDIC CRYOFIXATION SYSTEM

The *in situ* microfluidic cryofixation system is designed as a self-contained modular microscopy stage (Figure S1a). The design facilitates transfer of *in situ* cryofixed samples from the light microscopy experimental system to storage under liquid nitrogen (LN_2_). Following cryofixation of the sample within the microscope field of view, the entire assembly is transferred to a LN_2_ dewar. The assembly is submerged beneath LN_2_ during disassembly and recovery of the microfluidic device. The cryofixed sample within the microchannel remains in thermal contact with the copper heat sink during the transfer, preventing devitrification.

The main heater, used to warm the main PDMS microchannel during live imaging, is mounted onto a cold copper post. The cold post thermally connects a copper heat sink (40 mm wide x 40 mm long x 20 mm tall) with the main heater. During the experiment, the copper heat sink is partially submerged in a LN_2_ bath. The heat generated by the main heater is dissipated by the cold post, which limits the heater element to a 0.6 µm x 300 µm wide x 4 mm long NiCr strip. The microfluidic device is aligned such that the PDMS microchannel is in contact with the NiCr resistive heater (Figure S1b). The view in Figure S1b is generated by cutting the assembly along the red dashed-line in Figure S1a.

A unique feature of this microfluidic-based cryofixation system is that fluid flow remains possible until the moment of cryofixation. To achieve this aim, heat transfer is optimized to the full fluidic network consisting of three main components: macroscale fluidic channels, a silicon injector chip (green in Figure S1), and the main PDMS microchannel that houses the sample of interest. While the main heater warms the PDMS microchannel, three auxillary heaters warm the remainder of the fluidic network. One auxillary heater mounted on top of the silicon injector chip warms the bypass channels used to inject the sample into the PDMS microchannel. Fluidic connections to the silicon injector chip are made through 0.8 mm drilled holes in an actively heated metallic manifold. All external tubes connecting to the manifold are thermally insulated. During live imaging, a dry box encloses the entire assembly, including the light microscope objective, to prevent obstruction of the optical path by frost formation. The LN_2_ bath that cools the copper heat sink is the source of the nitrogen gas. The third auxillary heater thermally isolates the working fluidic system from the cold LN_2_ vapor that rises from the LN_2_ bath.

**Figure S1**. a.) The fully assembled *in situ* microfluidic cryofixation system. The system is designed as a self-contained, modular microscopy stage. The design allows transfer of the cryofixed sample from the light microscopy experimental setup to liquid nitrogen storage without devitrifying the sample. b.) The PDMS microchannel is mounted on a microfabricated NiCr resistive heater. The NiCr maintains the contents of the channel at room temperature during live imaging. c.) The layout of the PDMS microchannel and PDMS micropost array. The micropost array reduces the thermal mass of the system, as discussed in the main text, while providing mechanical stability to prevent excessive loading of the microchannel walls. Zigzag trapping structures preserve the *C. elegans* in the microscope field of view during live imaging.

Figure S1c shows the design of the PDMS microchannel. As discussed previously^1^, zig-zag trapping structures incorporated into the microchannel preserve the *C. elegans* in the microscope field of view during live imaging. Here, the location of the cryofixed nematode was determined from light microscopy images and compared to the known channel geometry. The FIB milling location was then determined by measuring the relative distance along the protruding microchannel with the SEM. As discussed in the main manuscript, in future work we plan to adapt previously reported procedures^2^ in order to automatically localize the sample within the microchannel.

CRYO-FIB MILLING VOLUME


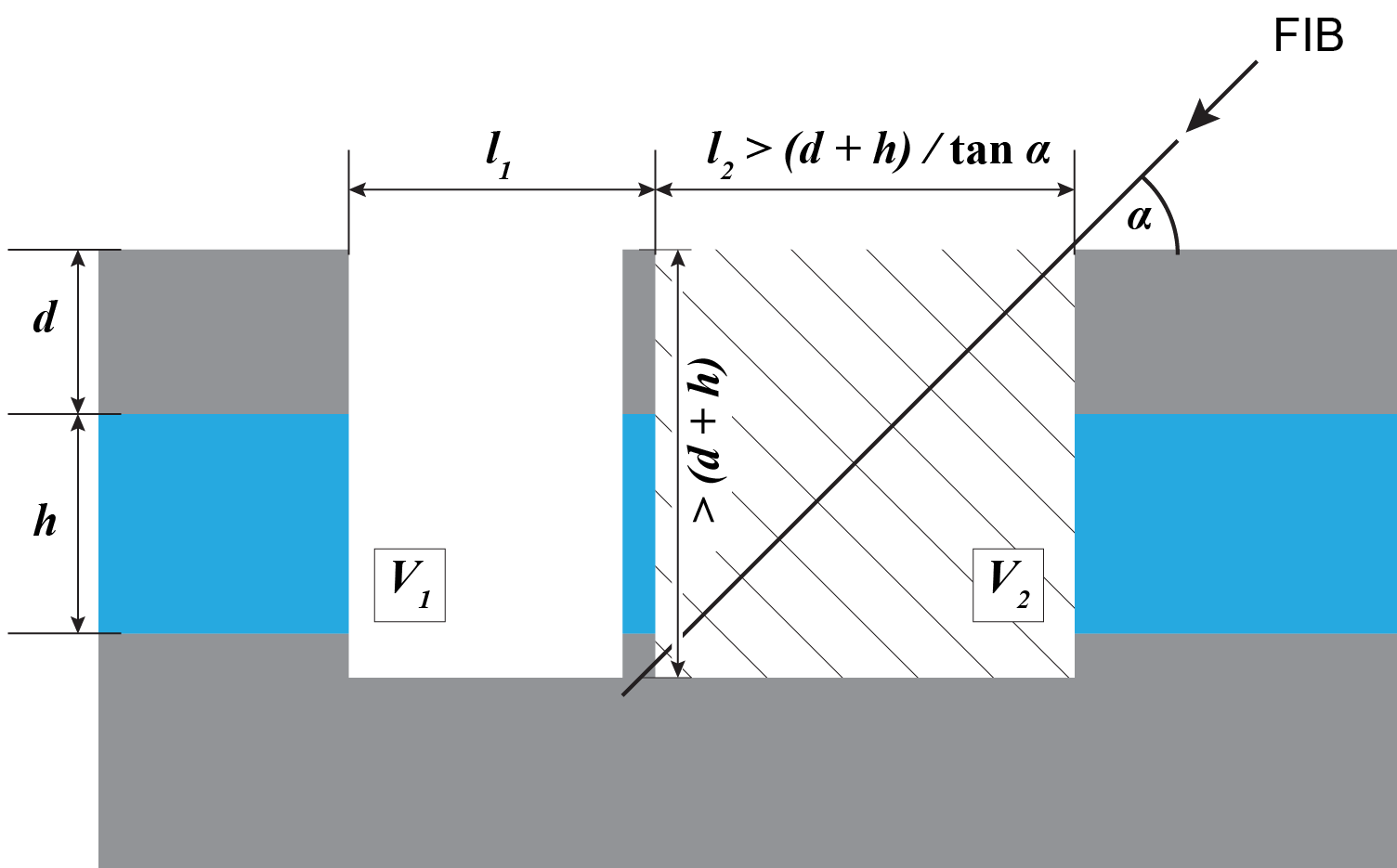


**Figure S2**. Geometry for cryo-FIB milling and undercutting of lamellae. The microfluidic channel is shown in blue. The top and bottom walls of the channel are made of PDMS (shown in grey). The minimum milling volume of the second trench, *V_2_*, necessary to release the lamella by undercutting is determined by the channel height *h*, the lid thickness *d*, the trench length *l_2_*, and the trench width *w* (not labeled, direction perpendicular to the drawing plane). The minimization of *d* by the new fabrication process described in this work has a strong influence on the milling time due to the relatively slow ablation rate of PDMS.

After defining a lamella by milling two trenches perpendicular to the surface of the device, the lamella is undercut at an oblique angle. The undercut sets a lower limit for the volume of material that must be removed by the ion beam for the second trench ($V_{2}$). Undercutting requires that one of the trenches must be long enough (dimension *l_2_* in schematic) to provide an unobstructed line-of-sight to the bottom of the lamella.

Therefore, the minimum milling volume increases linearly with the depth for the first trench

$${V_{1}>l}_{1}w\left( d+h \right)$$

and quadratically with the depth for the second trench:

$$V_{2}>\frac{{w\left( d+h \right)}^{2}}{\tan\alpha}.$$

Example parameters for full sample tilt ($\alpha={38}^{\circ}$) illustrating the significance of minimizing the PDMS lid thickness *d* are given in Table S1. It should be noted that the linear scaling of $V_{1}$ with (*d+h*) only applies with relatively small deviations in milling depth. As milling depth increases, the length (*l_1_*) must be extended to provide enough clearance for material sputtering.

**Table S1**. Approximate milling volume for a trench to enable the release of a thin lamella at an angle α = 38°. As PDMS has the slowest milling rate in the system, reducing the thickness of the PDMS layer from 15 to 3 µm reduces the rate-limiting volume *V*_PDMS_ by nearly 10x, which leads to a significant reduction of the total milling time.

| Parameter | Thin PDMS device | Thick PDMS device |
| --- | --- | --- |
| *d* | 3 µm | 15 µm |
| *h* | 20 µm | 20 µm |
| *w* | 20 µm | 20 µm |
| *l_1_* | 10 µm | 15 µm |
| *V_PDMS1_* | 600 µm^3^ | 4500 µm^3^ |
| *V_PDMS2_* | 1700 µm^3^ | 13400 µm^3^ |
| ***V_PDMS_*** | **2300 µm^3^** | **17900 µm^3^** |
| *V_total_* | 18100 µm^3^ | 41900 µm^3^ |

Fabrication OF PDMS Microchannels with <5 µm thick bottom walls

To achieve a bottom wall thickness of 3 µm or less, fabrication protocols that eliminate manual handling of fragile, ultra-thin PDMS layers were developed. An SU8 mold was first fabricated on a clean, dehydrated silicon handle wafer. The wafer was dehydrated at 200°C for 2 hours in a convection oven and then treated with an air plasma for 5 minutes. SU8 was spin-coated onto the silicon substrate (Figure S3). The first layer of the mold sets the height of the microchannel, here nominally 20 µm. The first SU8 layer was exposed to UV light in the channel pattern. A second layer was then spun onto the wafer to form the structural layer for the microfluidic posts. Microfluidic posts serve as the negative for fluidic through-holes. After a second exposure, the SU8 was developed to form a two-layer structure. A 100 nm anti-adhesion Parylene layer was deposited onto the mold using a SCS LabCoter 2 Parylene Deposition System.

To improve time efficiency, the process was designed to fabricate a full wafer of PDMS microchannels (28 in total) simultaneously. Towards this goal, a PDMS (Dow Corning, SYLGARD^TM^ 184) handling frame was formed by pouring uncured 10:1 PDMS between two silane-coated (Alfa Aesar, 1H,1H,2H,2H-Perfluorooctyltrichlorosilane, 97%) silicon wafers (step not shown in Figure S3). The distance between the wafers was set by 2 mm thick spacers. After curing, access windows for each microchannel were cut into the PDMS puck with a CO_2_ laser (Universal Laser Systems VLS4.60). The laser cut PDMS was sonicated (VWR International USC300th Ultrasonic Bath) in IPA for 20 minutes to remove surface particles.

PDMS was mixed with a 4:1 monomer to curing agent ratio, degassed, and spun over the SU8 master. The PDMS was partially cured on the SU8 mold in a convection oven at 60°C for 14 minutes. The PDMS handling frame was then aligned and brought into contact with the partially cured PDMS layer. The wafer was placed in the oven to finish curing at 80°C. The PDMS handling frame allowed all 28 devices on the wafer to be peeled from the master simultaneously. Following peeling, the microchannels suspended on the handling frame are still open at the bottom and bounded by three walls: the two sidewalls and the top wall with fluidic through holes.

To fabricate the thin-film PDMS bottom, a sacrificial layer was first spun onto a second silicon wafer. In this case, the sacrificial layer was positive tone photoresist (AZ 1518). Directly after spin-coating the AZ1518 (i.e. no soft-bake), a 10:1 PDMS layer was spun on top of the positive tone photoresist. The PDMS was fully cured at 80°C for 30 minutes.

The suspended microfluidic devices were bonded to the thin layer of PDMS (bottom wall) via oxygen plasma bonding to form sealed channels. The sacrificial layer was then dissolved in acetone to release the thin-bottom microfluidic devices from the silicon handle wafer. At this point, the PDMS foil was trimmed to a 5 mm x 5 mm square with a CO_2_ laser (Universal Laser Systems VLS4.60) and sonicated in IPA. The cleaned foils were then bonded to silicon injector chips to form individual microfluidic devices. The channel bottom wall thickness measured 2.9 ± 0.2 µm for samples cryofixed for cryo-EM. Microchannels with bottoms walls as thin as 0.8 ± 0.1 µm can be fabricated reliably with this method.

**Figure S3**. Schematic of the PDMS microchannel fabrication process described in the main text.

COOLING RATE CHARACTERIZATION

The cooling rate was measured using a solution of DI water with 1% (m/m) Rhodamine B isothiocyanate–Dextran. Rhodamine B is a commonly used probe for non-contact temperature measurements as its quantum yield, and subsequently fluorescence intensity, increases as the media cools^3–5^. One recent report demonstrated Rhodamine B/DI water solution as a probe for the solid‑liquid phase transition. The fluorescence intensity of the Rhodamine B/DI water solution increased as the solution approached the freezing point, with an intensity drop following solidification^6^.

A similar effect was observed during slow cooling of our *in situ* microfluidic cryofixation system. After initial calibration of the assembly to ~25 °C according to previously reported procedures^1^, the heater power was slowly decreased corresponding to 1 °C steps in the channel temperature. Fluorescence intensity increased until reaching a maximum at ‑21 °C ± 4° C, and subsequently dropped after further decreasing the channel temperature by another 1 °C. Channel freezing was confirmed through three metrics (i) bright field imaging indicated the channel was rendered partially opaque due to ice crystallization in the channel^7,8^, (ii) light scattering and/or dye precipitation in fluorescence images due to ice crystallization in the channel, and (iii) fluid flow through the microchannel was no longer possible. Based on these metrics, the channel solidified immediately following the maximum of fluorescence intensity. Figure S4 shows bright field and fluorescence images of the microchannel at room temperature and immediately following freezing.

Based on the known temperature-dependence of Rhodamine B fluorescence intensity, we measured the time required for the temperature of the channel to drop from the working temperature (25° C) to ~ ‑21 °C during fast freezing (cryofixation). As discussed in the main manuscript, this measurement provides an estimate of the initial cooling rate averaged over the channel depth. The fluorescence maximum was reached in 2.4 ± 0.4 ms for a 2.9 µm thick PDMS bottom wall. Figure S5 shows a representative plot for *in situ* cryofixation for a microchannel with a 0.8 µm thick bottom wall. The contents solidified in 1.7 ± 0.3 ms, or ~1.5x faster than freezing in the device with a 3 µm thick bottom.

**Figure S4**. A representative plot of Rhodamine B fluorescence intensity during slow cooling in a microfluidic channel. The fluorescence intensity drops following solidification of the channel contents (‑21 °C ± 4 °C). Bright field images and fluorescence images show the microchannel at room temperature and immediately following freezing as indicated above.

**Figure S5**. *In situ* microfluidic cryofixation of Rhodamine B/DI water solution in a microchannel with a 0.8 µm thick bottom wall. The fluorescence maximum is reached in 1.7 ± 0.3 ms, or ~1.5x faster than freezing in the device with a 2.9 µm thick bottom.

In the case of *in situ* cryofixation, fluorescence intensity decays with time after reaching an initial maximum (see Figure 3). This intensity decay, however, does not correspond to a temperature change within the microfluidic environment, but rather depends on photophysical effects of the cryofixed dye solution. The maximum fluorescence intensity value is recovered by briefly interrupting the fluorophore excitation. Approximate decay time and initial maximum intensity values are consistent across exposure cycles of the cryofixed solution (Figure S6). Unlike photobleaching at room temperature, dark state population is reversible, which is consistent with our findings. The observed fluorescence decay following cryofixation is likely caused by long-lived electronic dark states of Rhodamine B at cryogenic temperatures^9,10^.

**Figure S6**. In the case of *in situ* cryofixation, fluorescence intensity decays with time after reaching an initial maximum as a function of the excitation source intensity. The excitation source shutter was manually opened and closed three times following cryofixation of Rhodamine/DI water solution. The intensity maximum was recovered each time after briefly interrupting exposure to the excitation source. Approximate maximum intensity values and decay time are consistent across multiple exposure cycles of the cryofixed solution.

CRYO ELECTRON TOMOGRAPHY

Figure S7 shows three different slices through the tomographic volume taken within a *C. elegans* that was cryofixed with the *in situ* microfluidic method. The images from the tilt-series used in the volume reconstruction are given in Figure S8. The data shows no evidence of ice crystallization.


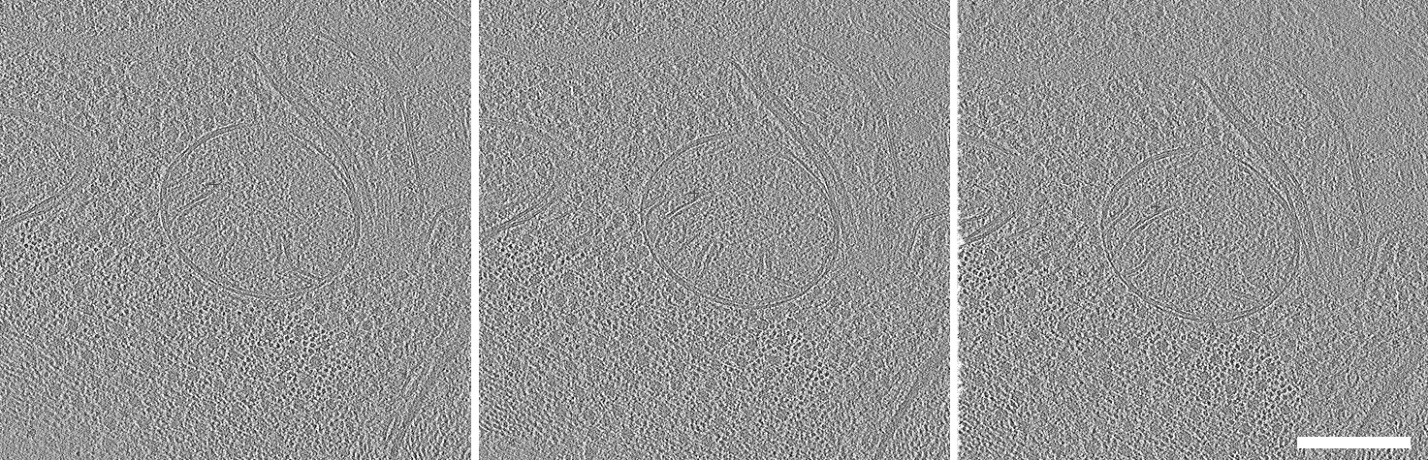


**Figure S7**. Three different slices through the tomographic volume exemplary of the high quality and resolution of the whole tomogram. Scale bar: 200 nm.


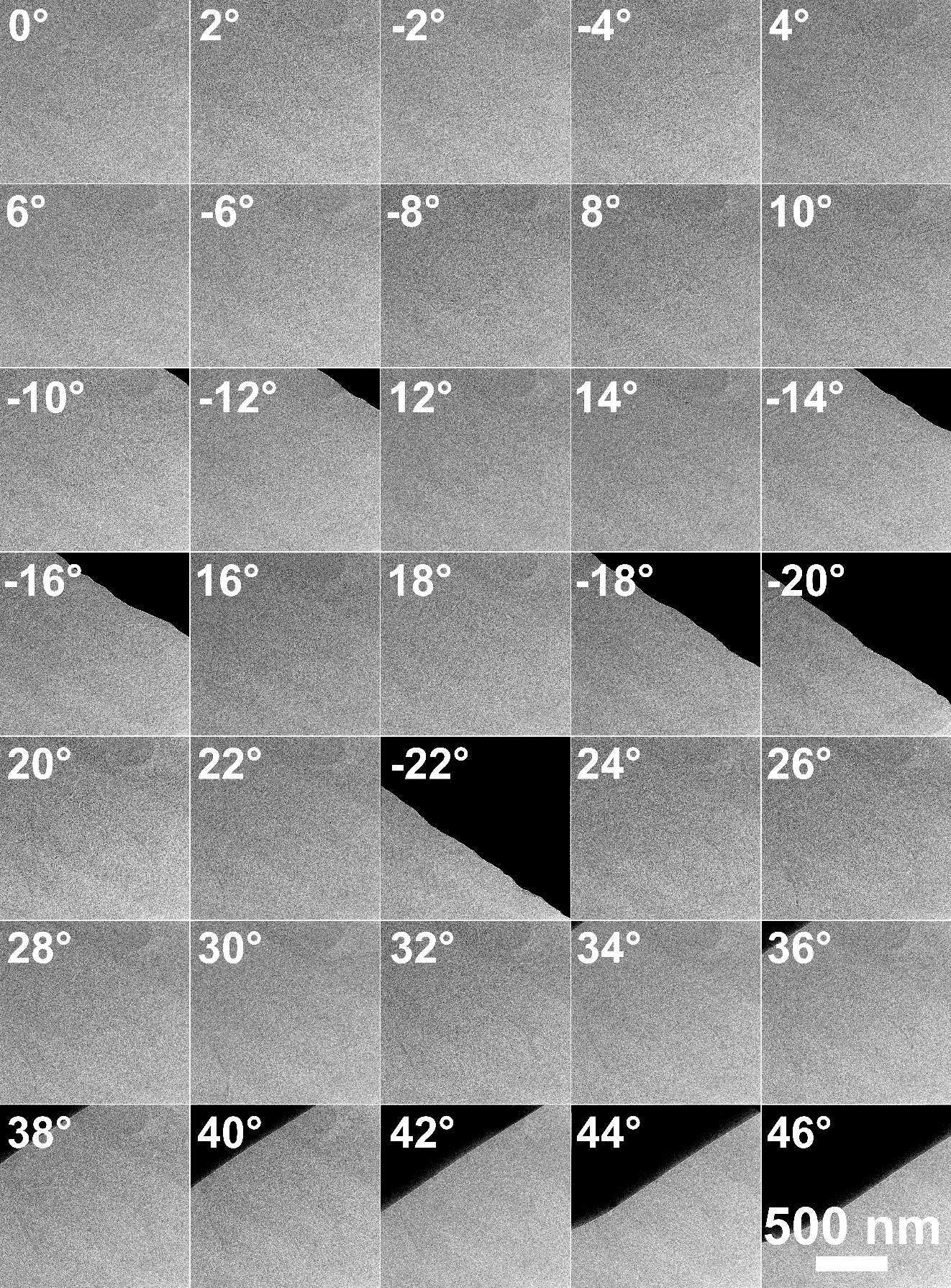


**Figure S8**. Tilt series for tomographic reconstruction in Figure 5 (main manuscript) and Figure S7. Each image is the drift-corrected sum of a direct-detection exposure frame stack. Black areas are shadows of the grid at high tilts as the volume of interest was close to the lamella edge. No diffraction contrast from crystalline ice is visible at any tilt.

CRYOPROTECTANT SCREENING

Cryoprotectants reduce the necessary cooling rate for sample ultrastructure preservation, as discussed in the main text. High concentrations of cryoprotectants, however, can have a detrimental effect on sample viability, where the critical concentration depends on both the type of cryoprotectant and specimen of interest. We performed a survival assay to guide selection of cryoprotectants for our *C. elegans* studies.

A *C. elegans* toxicity screening was performed for eight common cryoprotectants each with a concentration of 10% (m/v) in M9 and 20% (m/v) in M9. Cryoprotectants can be separated into two groups based on their chemical properties: those which diffuse across cellular membranes (penetrating) and those which do not (non-penetrating). Of the cryoprotectants tested, four were non- or minimally penetrating cryoprotectants (trehalose, sucrose, glucose, and PEG) and four were penetrating (glycerol, methanol, ethanol, and DMSO). Freshly hatched L1 larvae (*n*≥30) were manually picked from NGM plates and transferred into 12 well plates containing either M9 or an M9 based cryoprotectant solution. The plates were kept at a temperature of 20 °C throughout the assay. Figure S7a and S7b show the survival percentage following a 24 hour and 48 hour incubation period, respectively.

**Figure S9.** Survival assay for *C. elegans* incubated in either M9 or M9 based cryoprotectant solution for a.) 24 hours and b.) 48 hours. Cryoprotectant concentration is given in units of (% m/v). The sample size was ~35 nematodes at the start of the assay per condition.

The survival rate for the control sample (M9 without cryoprotectant) was 97% ± 3% after 24 hours and 94% ± 4% after 48 hours. Using a t-test with a 95% confidence interval there is a statistically significant difference between the survival rate of the control sample and all of the samples that were exposed to cryoprotectant. However, following 24 hours the survival rate exceeded 85% for 7 of the 16 cryoprotectant solutions; 10% trehalose, 20% trehalose, 10% sucrose, 20% sucrose, 10% glucose, 10% methanol, and 10% glycerol.

Of the non‑penetrating solutions, only the survival rate of *C. elegans* incubated in 10% trehalose remains above 90% after 48 hours. Notably, *C. elegans* incubated in the higher concentration of trehalose (20%) had a survival rate of 74% following 48 hours whereas exposure to the higher concentration of sucrose results in a 4% survival rate. All worms incubated in the higher concentration (20%) of glucose died in less than 24 hours.

In the case of the penetrating cryoprotectants, 10% (m/v) glycerol and 10% (m/v) methanol were the only two cryoprotectants with survival rates exceeding 85% after 24 hours (86% and 96% respectively). This margin changes significantly following 48 hours of incubation with a 70% survival rate for 10% (m/v) glycerol and only a 7% survival rate for 10% (m/v) methanol.

**Body Bend Assay**

In addition to animal survival, the lateral movement capacity of *C. elegans* in solution was quantified as a marker for the physiological health of the animal. This method is a well-established assay for studying the effects of drugs or mutations on the motility of *C. elegans*.^11,12,13^  Thrashings were counted manually for 15 seconds per animal using a dissection microscope 24 and 48 hours after transfer to the solutions. A reciprocating motion at the body midline was counted as one body bend. Animals that were not moving, due to either death or quiescence, were omitted from the assay.

For the surviving specimens, no statistically significant difference in number of body bends was observed between 10% trehalose, 20 % trehalose, and the M9 control group after incubation of 24 or 48 hours. There was a statistically significant difference between the lateral movement capacity of *C. elegans* exposed to 10% (m/v) glycerol compared to the M9 control group (Figure S8). The number of body bends after 24 hours for 10% (m/v) glycerol, however, remained within one standard deviation compared to the M9 sample group. Based on the results of our survival and body bend assays, 10% (m/v) trehalose and 10% (m/v) glycerol were selected as the non-penetrating and penetrating cryoprotectants respectively for *in situ* microfluidic cryofixation experiments. In this work we elected to use only a penetrating cryoprotectant (10% (m/v) glycerol), as in our previously reported EM images from room-temperature electron microscopy^1^.

**Figure S10.** Body bend assay for *C. elegans* incubated in either M9 or M9 + cryoprotectant solution for a.) 24 hours and b.) 48 hours. Cryoprotectant concentration is given in units of (% m/v). *C. elegans* that were not moving, due to either death or quiescence, were omitted from the assay.

SUPPORTING INFORMATION REFERENCES

1 M. Fuest, G. M. Nocera, M. M. Modena, D. Riedel, Y. X. Mejia and T. P. Burg, *J. Microsc.*, 2018, **272**, 87–95.

2 J. Arnold, J. Mahamid, V. Lucic, A. de Marco, J.-J. Fernandez, T. Laugks, T. Mayer, A. A. Hyman, W. Baumeister and J. M. Plitzko, *Biophys. J.*, 2016, **110**, 860–869.

3 D. Ross, M. Gaitan and L. E. Locascio, *Anal. Chem.*, 2001, **73**, 4117–4123.

4 D. Erickson, D. Sinton and D. Li, *Lab Chip*, 2003, **3**, 141–149.

5 T. Karstens and K. Kobs, *J. Phys. Chem*, 1980, **84**, 1871–1872.

6 I. Shishkin, T. Alon, R. Dagan and P. Ginzburg, *MRS Adv.*, 2017, **2**, 2391–2399.

7 J. B. Hopkins, R. Badeau, M. Warkentin and R. E. Thorne, *Cryobiology*, 2012, **65**, 169–178.

8 M. Warkentin, J. P. Sethna and R. E. Thorne, *Phys. Rev. Lett.*, 2013, **110**, 015703.

9 R. Kaufmann, C. Hagen and K. Grünewald, *Curr. Opin. Chem. Biol.*, 2014, **20**, 86–91.

10 S. Weisenburger, D. Boening, B. Schomburg, K. Giller, S. Becker, C. Griesinger and V. Sandoghdar, *Nat. Methods*, 2017, **14**, 141–144.

11 S. D. Buckingham and D. B. Sattelle, *BMC Neurosci.*, 2009, **10**, 84.

12 A. Bansal, L. J. Zhu, K. Yen and H. A. Tissenbaum, *Proc. Natl. Acad. Sci. U. S. A.*, 2015, **112**, E277-286.

13 J. H. Hahm, S. Kim, R. DiLoreto, C. Shi, S. J. V Lee, C. T. Murphy and H. G. Nam, *Nat. Commun.*, 2015, **6**, 8919.
